# Toll-like receptor 4 (TLR4) expression in human and murine pancreatic beta-cells affects cell viability and insulin homeostasis

**DOI:** 10.1186/1471-2172-12-18

**Published:** 2011-02-28

**Authors:** Humberto M Garay-Malpartida, Roberta F Mourão, Marluce Mantovani, Icaro A Santos, Mari C Sogayar, Anna C Goldberg

**Affiliations:** 1 Núcleo de Terapia Celular e Molecular, Universidade de São Paulo, São Paulo, Brasil; 2 Escola de Artes, Ciências e Humanidades, Universidade de São Paulo, São Paulo, Brasil; 3 Instituto de Química, Departamento de Bioquímica, Universidade de São Paulo, São Paulo, Brasil; 4 Instituto de Investigação em Imunologia (iii), Institutos Nacionais de Ciência e Tecnologia, Brasil; 5 Hospital Israelita Albert Einstein, São Paulo, Brasil

## Abstract

**Background:**

Toll-like receptor 4 (TLR4) is widely recognized as an essential element in the triggering of innate immunity, binding pathogen-associated molecules such as Lipopolysaccharide (LPS), and in initiating a cascade of pro-inflammatory events. Evidence for TLR4 expression in non-immune cells, including pancreatic β-cells, has been shown, but, the functional role of TLR4 in the physiology of human pancreatic β-cells is still to be clearly established. We investigated whether TLR4 is present in β-cells purified from freshly isolated human islets and confirmed the results using MIN6 mouse insulinoma cells, by analyzing the effects of TLR4 expression on cell viability and insulin homeostasis.

**Results:**

CD11b positive macrophages were practically absent from isolated human islets obtained from non-diabetic brain-dead donors, and TLR4 mRNA and cell surface expression were restricted to β-cells. A significant loss of cell viability was observed in these β-cells indicating a possible relationship with TLR4 expression. Monitoring gene expression in β-cells exposed for 48h to the prototypical TLR4 ligand LPS showed a concentration-dependent increase in TLR4 and CD14 transcripts and decreased insulin content and secretion. TLR4-positive MIN6 cells were also LPS-responsive, increasing TLR4 and CD14 mRNA levels and decreasing cell viability and insulin content.

**Conclusions:**

Taken together, our data indicate a novel function for TLR4 as a molecule capable of altering homeostasis of pancreatic β-cells.

## Background

Islets are small organ-like structures, which are rich in vasculature and are predominantly constituted of insulin-secreting β-cells and glucagon-producing α-cells. As the main target of autoimmunity that results in type 1 diabetes, β-cells have been the subject of numerous studies aiming to identify the mechanisms which cause the massive cell death that ultimately leads to overt disease. In the setting of autoimmunity, β-cells respond to the onslaught of proinflammatory cytokines produced by immune cells, such as interleukin (IL)-1β, tumor necrosis factor (TNF) -α and interferon- γ, which trigger the NF-κB pathway and promote transcription of genes that ultimately cause β-cell dysfunction and cell death [1].

A promising treatment for type 1 diabetes is islet cell transplantation [2]. To this end, pancreata from brain-dead non-diabetic donors are selected and islets are harvested and purified in clean room facilities for infusion into the portal vein of diabetic patients. However, the isolation procedure employed to obtain purified islets is time-consuming and frequently fails to produce sufficient good quality islets to enable a successful engraftment. The low yield during the isolation procedure can be credited, at least in part, to previously identified factors present in the organ donors, such as: advanced age, cause of brain death, episodes of anoxia, hypertension, use of vasopressors, which cause ischemia, poor glycemic control, and extended periods in the Intensive Care Unit [3,4]. In addition, studies in an experimental model showed that brain death triggers an early increase in serum proinflammatory cytokines, such as TNF-α, IL-1, and IL-6 and that their transcription, together with that of the chemokine Monocyte Chemotactic Protein-1 (MCP-1), are increased in the pancreas [5,6].

Inherent difficulties in separating islets from the surrounding acinar tissue may also contribute to a lower islet recovery [7-9], and a common feature resulting from the isolation procedure will be a certain amount of cell death and loss of insulin content; both being hallmarks of low-quality harvested islets.

In the past two decades, Toll-like receptors (TLRs) have been progressively acknowledged as crucial pathogen recognition receptors, playing major roles in innate responses, as well as in triggering adaptive immunity [10]. The manifold properties of TLRs have been mainly identified in immune cells, especially macrophages and dendritic cells [11]. The most extensively studied receptor is TLR4, which is the receptor for Gram-negative bacterial endotoxins, collectively known as LPS. Control of TLR4 activation involves glycosylphosphatidylinositol (GPI)-anchored CD14, MD-2, and the Lipopolysaccharide-binding protein (LPB). LBP binds to the lipid A moiety of LPS and transfers LPS to CD14, which guarantees and optimizes signaling through the TLR4 complex [12].

Besides being found in antigen-presenting cells, several TLRs, and more specifically, TLR4, have been identified in other cell types, such as endothelial cells [13,14], myocytes [15], thyroid cells [16], endometrial cells [17], mesangial cells [18], and adipocytes [19]. In each of these cases, TLRs participate in responses associated to stress and disease. TLR4 has also been identified in human β-cells and β-cell lines such as HP62 [20]. In 2003, using immunofluorescence-based methods, whole islets and the SV40-transformed HP62 β-cell line were shown to express TLR4 and CD14. However, the expression was not restricted to β-cells, since glucagon-secreting α-cells and even ductal cells were also positive.

In the present study, we investigated whether "ex-vivo" expression of TLR4 occurs in β-cells from islets harvested from brain dead donors, and the effects of TLR4 activation on insulin production and secretion, and on cell viability.

## Results

### Islets harvested from brain dead donors display variable expression levels of TLR4

Analysis of gene expression at the mRNA level, by quantitative RT-PCR, in freshly isolated human islets or in non- adherent islet cultures, confirmed the presence of TLR4 transcripts. In 11 out of 12 islet cell preparations, from different pancreata, analyzed immediately after the isolation procedure, variable levels of TLR4 gene expression were observed, which persisted for up to 48h in culture. No association was observed between TLR4 expression levels and glucose-induced insulin secretion in purified islets, cold ischemia time (time lapse between organ procurement and the beginning of the isolation procedure) or other donor variables, such as donor age, days in the Intensive Care Unit or the presence of infection (data not shown). However, when the relative mRNA expression was measured at 0, 24, and 48h, the Pearson coefficient test (-0.9 <*r *> 0.9, p < 0.05) revealed a positive correlation between TLR4 and well known pro-inflammatory molecules involved in β-cell death, namely caspase-1, MCP-1, IL-1 receptor antagonist, and Fas (CD95) gene expression levels. This correlation was particularly evident in pancreatic islets obtained after low yield islet isolation procedures, in which islets displayed low cell viability, as measured by the live/dead fluorescence method, even though the relative mRNA levels differed in islets from one pancreas to another (Table 1).

**Table 1:** Outcomes of islet isolation from pancreata used in this study

**Pre-isolation**				**Post-isolation**				
	
Isolation procedure	Age	Sex	Glycemic levels	Total IEQ^(1)^	Purity^(2)^	Viability^(3)^	TLR4 expression	
	
			(mg/dL)				mRNA^(4)^	Correlation^(5)^
	
PHum1	42	M	186	143,000	70%	80%	Yes	No
PHum2	52	F	ND	116,000	80%	80%	Yes	No
PHum3	42	M	109	50,000	50%	70%	Yes	Yes
PHum4	41	M	149	73,000	70%	60%	Yes	No
PHum5	56	F	140	66,160	60%	50%	Yes	Yes
PHum6	55	M	143	204,458	90%	90%	Yes	No
PHum7	54	M	164	23,000	40%	40%	Yes	Yes
PHum8	53	F	113	60,000	70%	70%	Yes	Yes
PHum9	55	F	193	93,333	80%	80%	Yes	No
PHum10	35	M	162	555,125	90%	90%	No	No
PHum11	55	F	293	380,233	80%	90%	Yes	No
PHum12	49	F	93	48,000	60%	50%	Yes	Yes

(1) Each IEQ (islet equivalent) represents one islet 150 μm in diameter, (2) Purity is defined by percentage of Dithizone-positive cells (beta cells) in the islet preparations, (3) Viability as measured by live/dead fluorescence, (4) Relative TLR4 mRNA expression levels are variable and this column indicates only presence (Yes) or absence (No) of detectable levels of TLR4 mRNA at any time point, (5) Correlation was obtained from time-course analysis of mRNA expression of TLR4 and β-cell death-associated molecules by the Pearson coefficient test. The presence (Yes) or absence (No) corresponding to a significant positive or negative linear correlation (-0.9 <*r *> 0.9, *p *> 0.05) is indicated in this column.

### TLR4 expression observed on the surface of human pancreatic islets is mainly restricted to β-cells

We then investigated whether the TLR4 gene expression observed was restricted to functional endocrine islets cells or due to infiltrating macrophages and dendritic cells present within the islets.

**Figure 1:**
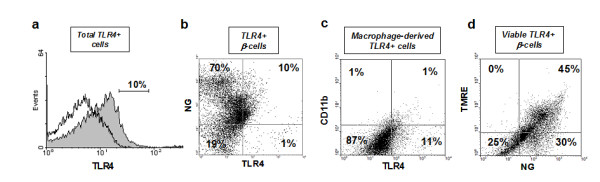
**Flow cytometric analysis of TLR4 expression in human islet cells**. Free-floating 8-12 day islet cultures (500-1000 IEQ) were dissociated into single-cell suspensions and assessed by flow cytometry. A representative image from three independent experiments is shown. **(a) **Histogram compares total TLR4-positive cells (gray) with isotype-matched goat anti-rabbit IgG control for TLR4 staining (no color). **(b) **The right upper quadrant of the dot plot shows the TLR4 positive β-cell population, double-stained for Newport Green (NG) and TLR4-APC conjugate, **(c) **The right upper quadrant of the dot plot shows TLR4-positive macrophage-derived cells (non β-cells) double stained with TLR4-APC conjugate and CD11b-PE conjugate, **(d) **Tetramethylrhodamine (TMRE) and NG double staining marks viable β-cells.

To confirm which cell types were expressing the functional TLR4 protein, triple staining with Newport Green (NG), which is specific for Zn-rich insulin granules [21] anti-TLR4-APC, and anti-CD11b-PE antibodies, was performed for flow cytometric analysis in isolated cells obtained after treatment with Accutase. As observed with mRNA transcripts, islets showed variable levels of TLR4 expression on the cell surface (11.3 ± 4% of total cells, n = 3) (Figure 1a), which was almost exclusively present in insulin-producing β-cells (84.7 ± 3% of TLR4-positive cells) (Figure 1b). The percentage of CD11b+ cells was very low, but consistently TLR4 positive (9.2 ± 2% of TLR4-positive cells) (Figure 1c). The fact that macrophages are present in a very low number is not surprising, considering that these islets were obtained from non-diabetic donors and non-infected pancreata, therefore, one would not expect to find infiltrating leukocytes.

### LPS-induced TLR4 expression in cultured β-cells leads to loss of cell viability

**Figure 2:**
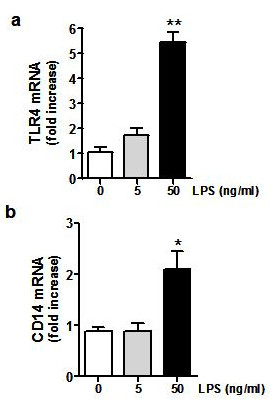
**LPS-induced TLR4 expression in β-cells**. 5 × 10^5 ^human β-cells from adherent cultures were treated with LPS (5 and 50 ng/mL) and analyzed after 48 h. Expression of **(a) ***TLR4 *and **(b) ***CD14 *mRNA is shown as mean ± SD (3 independent experiments) of relative fold increase in gene expression at 48 h, compared to control (untreated) cells. Statistically significant differences are shown as (*) *p *< 0.05 and (**) *p *< 0.005.

**Figure 3:**
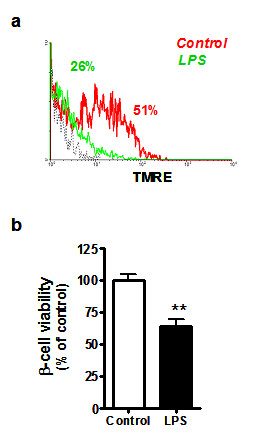
**Long-term exposure to LPS induces a loss of β-cell viability**. Under the same conditions described in the legend to Figure 2, cell viability was analyzed by flow cytometry using TMRE staining. **(a) **Histogram showing the percentage of TMRE-positive cells upon treatment with 50 ng/mL LPS (red line), untreated control cells (green line) and isotype-matched control for TLR4 staining (dotted black line). **(b) **Values represent the relative percentage of TMRE-positive β-cells. Data are expressed as mean ± SD for β-cell cultures derived from different pancreas donors (n = 7), incubated with or without LPS 50 ng/mL for 48 h and analyzed in triplicate. Statistically significant differences are indicated as (**) *p *< 0.005, compared to non-treated control cells.

To investigate whether TLR4 activation affects viability of the pancreatic β-cells, the response of adherent cell cultures to LPS was tested. Following a 48h exposure to different concentrations of LPS (0, 5, and 50 ng/mL), the islet cells showed a markedly enhanced mRNA expression of both TLR4 and the LPS-associated CD14 molecules, in a concentration-dependent manner (Figure 2a-b). After 48h the cells were harvested and analyzed by flow cytometry with anti-TLR4/NG/TMRE triple staining. A significant reduction of cell viability was observed (Figure 3a), reaching 36.2 ± 5% (n = 7, *p *< 0.01) at 48h (Figure 3b), revealing the involvement of the activated TLR4 signaling pathway in the mechanism of β-cell death.

### TLR4 expression is accompanied by decreased insulin synthesis and secretion

Since a recently reported study indirectly associated TLR4 to glucose-mediated insulin regulation in human peripheral blood monocytes [22], we hypothesized that this receptor could also be operating a negative regulatory mechanism in pancreatic β-cell insulin production and secretion.

**Figure 4:**
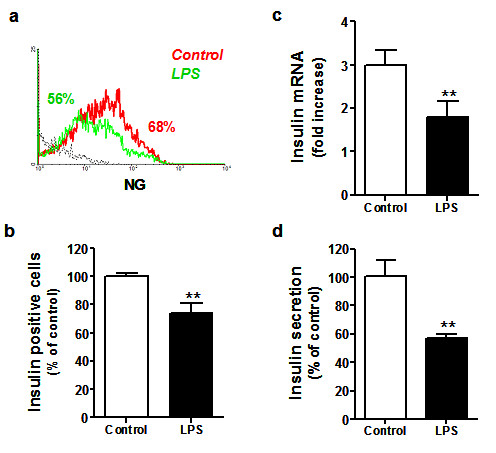
**Effects of LPS treatment on insulin synthesis and secretion by β-cells**. After 48 h of LPS stimulation, the effects on insulin homeostasis were measured by flow cytometry. **(a) **Representative histogram of percent NG-positive cells after treatment with LPS (red line), untreated or control cells (green line) and isotype-matched controls for the TLR4 staining (dotted black line). Data represent relative percentage of **(b) **NG-positive cells compared to control cells, expressed as mean ± SD for several β-cell cultures derived from different pancreas donors (n = 7), and analyzed in triplicate. **(c) **Insulin mRNA expression is shown as mean ± SD (3 independent experiments) of relative fold increase compared to control (untreated) cells. **(d) **Insulin secretion measured by chemiluminescence in β-cell cultures isolated from two different pancreata expressed as relative percentage of insulin secretion observed in control cells, and analyzed in triplicate (mean ± SD). Statistically significant differences are indicated as (**) *p *< 0.005, as compared to non-treated control cells.

As measured by NG-positive staining (Figure 4a), the relative proportion of insulin content in LPS-treated cells was reduced by 25.8 ± 6.5% (n = 7, *p *< 0.001), when compared with non-treated cells (Figure 4b). Under the same conditions, we observed a discrete, but significant, decrease of insulin mRNA expression (Figure 4c). In agreement with these data, a 43.5 ± 3.7% (n = 7, *p *< 0.0001) decrease in insulin secretion was observed in β-cells treated with LPS (Figure 4d).

### The effects of LPS-induced TLR4 expression in mouse MIN-6 cells closely resemble those observed in cultured human islets

**Figure 5:**
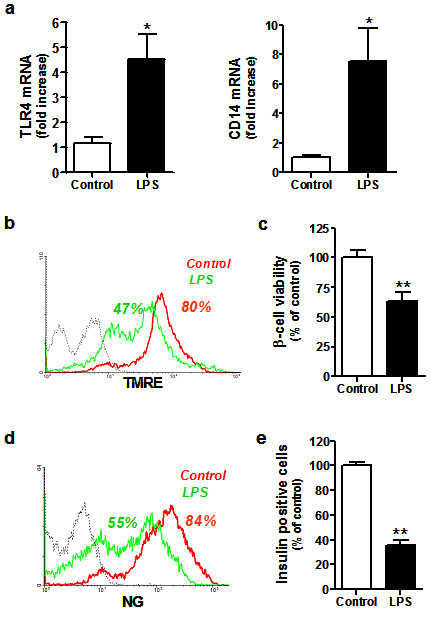
**Effects of LPS on TLR4 expression, cell viability and insulin synthesis in murine insulinoma cells**. 5 × 10^5 ^MIN-6 cells treated with LPS (50 ng/mL) were analyzed after 48 h for gene expression, cell viability and insulin production. **(a) ***TLR4 *and *CD14 *mRNA expression is shown as mean ± SD (n = 3) of relative fold increase compared to control (untreated) cells. Representative flow cytometry histogram of **(b) **percent TMRE-positive or **(d) **percent NG-positive cells treated with LPS (red line), untreated or control cells (green line) and isotype-matched control for TLR4 staining (dotted black line). Relative percentage of **(c) **TMRE positive or **(e) **NG positive cells expressed as mean ± SD (n = 3). In all cases, statistically significant differences are indicated as (*) *p *< 0.05 and (**) *p *< 0.005, compared to non-treated control cells.

In order to confirm the effects of TLR4 activation in β-cells, we assessed LPS-induced stimulation of TLR4 expression in MIN-6 mouse insulinoma cells. Under the same conditions used for human islet adherent cell cultures, we observed a 4.5 and 7.6 fold increase in both TLR4 and CD14 mRNA levels, respectively (Figure 5a), which were accompanied by a 35.9 ± 3% loss in cell viability (n = 7, *p *< 0.001) in response to LPS (50 ng/mL) in MIN-6 cells (Figure 5b-c). LPS was also capable of inducing a strong reduction of insulin content (74.6 ± 4.2%, n = 3), as measured by NG-positive staining (Figure 5d-e). Taken together, these data suggest that the mechanisms for cell death and regulation of insulin homeostasis can be modulated by TLR4 in human and murine LPS-stimulated β-cells.

## Discussion

In accordance with a growing literature showing that Toll-like receptors exhibit functions in the homeostasis of several types of non-immune cells [13-20], here we show that β-cells harvested from human non-diabetic brain-dead organ donors also express variable levels of TLR4. Our results indicate that there could be a role for TLR4 in β-cell homeostasis, possibly related to cell viability or insulin production.

In contrast with a previous study [20], we showed that LPS not only increases TLR4 expression and diminishes cell viability, but, also, leads to loss of β-cell insulin content. In both non-diabetic human and murine β-cells, TLR4 as well as CD14 transcripts were identified, with LPS (50 ng/mL) leading to a concentration-dependent increase in their gene expression levels, in parallel with lowered cell viability and decreased insulin content and mRNA transcription. These results are in agreement with a study showing that blocking TLR4 expression in murine islets by carbon monoxide treatment leads to increased survival of islet implants in an allogenic murine model [23]

In a recent study [24] with the BRIN BD11 rat clonal cell line, the authors showed decreased insulin secretion upon culturing in the presence of LPS, but no change in insulin content. The authors also observed a reversal of the effect on insulin secretion upon removal of LPS. The observed lack of change in insulin content may be explained by the fact that cells were incubated with 100 ng/mL LPS only for 24h, in contrast to our incubation period for up to 48h, but using half the dose of LPS. Indeed, in our study, we also observed a low effect upon insulin content when cells were incubated for 24h (data not shown). Taken together, these findings suggest that effects on β-cell insulin content and cell viability require more time to take place.

In addition to the classical activation, via pathogen-associated molecular patterns, TLR expression in monocytes has been shown to be regulated by glucose [22]. High glucose levels induced TLR2 and TLR4 expression while Mannitol did not, excluding an osmotic effect as the cause for observed results. Increased TLR4 expression occurred due to PKC-δ-mediated activation and stimulated p47Phox-dependent NADPH oxidase activity, establishing a link between high glucose, NF-κB-mediated inflammatory cytokine production, and TLR expression. In another study, with type 2 diabetic patients, in which TLR expression in mononuclear cells was monitored in response to low-dose insulin infusion, a further relationship between TLR2 and TLR4, and insulin homeostasis was identified [25]. Steady-state infusion during a short 4h period caused an increase of approximately 2.5 fold in insulin plasma levels while maintaining the patient's glycemia, and led to decreased TLR expression, which occurred independently of blood glucose levels. The effect of this short-term experiment, however, was more pronounced on TLR2 than on TLR4 expression. It is possible that increasing the period of infusion would have increased the effect on TLR4 expression.

Our study suggests a further link between insulin homeostasis, cell viability, and TLR4 expression. Although we did not measure apoptosis-related molecules, we used TMRE as an indirect marker for apoptosis. TMRE is a dye with a high degree of membrane permeability that accumulates in viable mitochondria, thereby marking only cells that are not undergoing apoptosis. In the presence of LPS-induced TLR4 expression, in both human and murine β-cells, insulin mRNA, insulin secretion and insulin content were diminished, and β-cell viability was decreased (from 100 to approximately 60%, Figure 3b).

Endogenous ligands for TLR4 have been described, most of which have been proven to exert their effect independently of an eventual experimental LPS contamination [26]. The list includes heat shock proteins 60 and gp96 [27,28], fibronectin type III extra domain A [29], hyaluronan [30], saturated fatty acids [15], advanced glycation end-products [31] and heme [32]. In other words, molecules produced or circulating during abnormal situations such as tissue damage and ischemia are capable of triggering TLR4- dependent pathways. These conditions are commonly present in brain-dead organ donors, being potential causes of engraftment failure as well as setting the conditions for inappropriate expression of TLR4 on non-immune cells. It remains to be seen how many of these potentially damaging factors are present before and during the long-lasting islet isolation procedures, and which, by favoring TLR4 expression, may further contribute to diminished insulin content and lower islet yield.

We can also envisage that increased glucose plasma levels not only induce insulin secretion, triggering insulin gene transcription and replacement of the intracellular stocks, but also increase surface expression of TLR4 molecules. The release of endogenous TLR4 ligands during tissue damage and inflammation followed by docking onto β-cells, could then result in loss of the ability of these β-cells to respond normally to glucose, contributing to the extra load of circulating plasma glucose and worsening of the glycemia control observed in response to TNF-α and adrenalin, as well as other hormones secreted under stressful situations.

## Conclusion

In conclusion, in the present study, we not only confirm the expression of TLR4 in pancreatic β-cells, but also suggest that its expression impacts upon β-cell viability and insulin synthesis and secretion, indicating that TLR4 should be included in the list of molecules affecting control of insulin homeostasis in β-cells.

## Methods

### Islet isolation and primary cell culture

Pancreata were removed from adult non-diabetic brain-dead donors (mean age 54 ± 5 years, n = 12) after *in situ *vascular perfusion with cold University of Wisconsin (UW) solution, in accordance with Brazilian regulations. The clinical trials were cleared by the local Institutional Ethics Committees at Universidade de São Paulo and Hospital Israelita Albert Einstein, Approval from the Brazilian National Ethical Committee (CONEP http://conselho.saude.gov.br/web_comissoes/conep/index.html) for pancreas procurement and human pancreatic islet isolation is registered under n° 4506. Pancreatic islets were isolated after ductal distension of the pancreas and tissue digestion with Liberase H1 (Roche Diagnostics, Indianapolis, IN) according to the automated method of Ricordi et al. [33]. Islet preparations exhibited 60-90% purity as determined by Dithizone staining. Islet cell viability was evaluated by the live/dead fluorescence, based on incorporation of either acridine orange by live cells or propidium iodide by dead cells, being usually greater than 60%. Upon isolation, islets were maintained in free-floating conditions in non-adherent T75 flasks with CMRL 1066 medium (Mediatech-Cellgro, Miami, FL) supplemented with 20% human albumin, 100 mM sodium piruvate, 30 mM vitamin E, 2.5 mM nicotinamide, and 0.2% ciprofloxacin. All cultures were maintained at 37°C, and 5% CO_2 _in a humidified incubator. Part of the islet preparation, that is 3000-5000 islet equivalents (IEQ), which correspond to approximately 3 × 10^5 ^cells/mL, was kept in non-adherent flasks and cultured, as described above, during 8-12 days. The remaining islets were transferred to T75 adherent culture flasks and grown in CMRL 1066 supplemented with 100 units/mL penicillin and 5% of fetal calf serum (FCS). After spontaneous dissociation with a 1g/L trypsin solution (Life Technologies Inc., Grand Island, NY) for 1 min at room temperature, 5 × 10^5 ^cells/well were plated in 6-well adherent dishes. At 70-80% confluence, cells were treated with the indicated concentrations of LPS 0-50 ng/mL (Ctlg # 62326, Sigma-Aldrich, IL) during 48 h. All human cell cultures were maintained in the absence of FCS to avoid interference from collected medium in the insulin measurements.

### Murine cell line

MIN-6 mouse insulinoma cells (a gift from Professor A.C. Boschero, UNICAMP, Campinas, Brazil) were cultured at 37°C in RPMI medium containing 5.6 mM glucose, 10% FCS, 100 U/mL penicillin, and 100 μg/mL streptomycin in a humidified atmosphere with 5% CO_2_. For the assays, 5 × 10^5 ^cells were plated per well in adherent 6-well dishes. At 70-80% confluence, cells were treated with the indicated concentrations of LPS (0-50 ng/mL) for different periods of time (0-48 h) with only a minimum (0.5%) of FBS added.

### Flow cytometry

Islets from free-floating cell cultures (500-1000 IEQ) were harvested by centrifugation at 280g for 5 min at 4°C, and dissociated into single-cell suspensions by treatment with Accutase (Innovative Cell Technologies, Temecula, CA) for 10-12 min at 37°C as previously described [34]. Adherent islet cells and MIN6 cell cultures were dissociated with a 1g/L trypsin solution for 1min at room temperature. In all cases, single cells were harvested by centrifugation (280 × g for 5 min at 4°C), washed twice with PBS and filtered through a 0.70 μm mesh nylon filter. Cells were resuspended in PBS at a concentration of 10^7 ^cells/mL and aliquots (10^4 ^cells/μl) were transferred to FACS tubes. To stain insulin positive cells, we used 1 μM Newport Green (NG) in PBS (Newport Green PDX^® ^acetoxymethyl ether, Molecular Probes, Eugene, OR). Cell viability was measured with Tetramethylrhodamine (TMRE) 100 nM (Molecular Probes, Eugene, OR) staining. In both assays, cells were incubated during 1 h at 37°C in the dark. To discriminate TLR4-positive viable β-cells, a triple staining with anti-TLR4-APC conjugate (eBioscience, San Diego, CA), NG and TMRE was performed during 15 min at 37°C, in the dark. TLR4-positive immune cells were identified by triple staining, achieved under the same conditions, but using anti-TLR4-APC conjugate, NG and anti-CD11b-PE conjugate (BD Biosciences, San Jose, CA). Stained cells (10,000-20,000 cells/sample) were analyzed with a FACSCalibur flow cytometer, using the CellQuest program (BD Biosciences, San Jose, CA). Unstained cells (auto-fluorescence) or goat anti-rabbit IgG (isotype-matched) were used as reference controls for each experiment. Data were analyzed with WinMDI software (Scripps, La Jolla, CA).

### Real-time quantitative RT-PCR (qRT-PCR)

Total RNA from 1,000-2,000 IEQ was extracted using Trizol^® ^reagent (Invitrogen, San Diego, CA), according to the manufacturer's instructions, and cDNA was synthesized from 2 μg of total RNA using SuperScript™ III Reverse Transcriptase kit (Invitrogen, San Diego, CA) with oligo dT primers. Real-time PCR was performed according to the Sybr^®^Green assay protocol (Applied Biosystems, Foster City, CA), using the Sequence Detector ABI PRISM 7500 (Perkin-Elmer/Applied Biosystems, Foster City, CA, USA). The gene-specific PCR primers were designed to span an intron with Primer3^® ^software (sequences available upon request). We used a 2-step amplification protocol with a denaturing temperature of 95°C and an annealing-extension temperature of 60°C. The relative gene expression was calculated from cycle threshold values (Ct) using the Pfaffl method [35]. Human or murine HPRT gene expression was used as an internal reference for each individual sample.

### Cumulative insulin secretion

Conditioned medium from cultures incubated for 48h in the presence or in the absence of 50 ng/mL LPS was collected and accumulated insulin release was quantified by the ELECSYS chemiluminescence assay, according to the manufacturer instructions (Roche Diagnostics, Indianapolis, IN). Results were normalized by total protein quantified by the Bradford method.

#### Statistical analysis

Data are presented as the mean ± standard deviation (SD). Each experiment was repeated at least three times with triplicate values within each group. Differences between means were analyzed by Student's t test. A p value < 0.05 was considered statistically significant. Analyses were performed using the Prism software version 4.0 (Graph Pad Software Inc., San Diego, CA).

## Authors' contributions

HMGM and ACG were responsible for the design and coordinated the present study. HMGM, IAS, RFM, and MM performed the experiments and analyzed the data. HMGM and ACG wrote the manuscript. MCS was responsible for providing the infrastructure, discussing the results and reviewing the manuscript. All authors read and approved the final manuscript.

## References

[B1] Cardozo AK Heimberg H Heremans Y Leeman R Kutlu B Kruhoffer M Orntoft T Eizirik DL A comprehensive analysis of cytokine-induced and nuclear factor-kappa B-dependent genes in primary rat pancreatic beta-cells J Biol Chem 2001 276 48879 86 10.1074/jbc.M108658200 11687580

[B2] Shapiro AM Ricordi C Hering BJ Auchincloss H Lindblad R Robertson RP Secchi A Brendel MD Berney T Brennan DC Cagliero E Alejandro R Ryan EA DiMercurio B Morel P Polonsky KS Reems JA Bretzel RG Bertuzzi F Froud T Kandaswamy R Sutherland DE Eisenbarth G Segal M Preiksaitis J Korbutt GS Barton FB Viviano L Seyfert-Margolis V Bluestone J Lakey JR International trial of the Edmonton protocol for islet transplantation N Engl J Med 2006 355 1318 30 10.1056/NEJMoa061267 17005949

[B3] O'Gorman D Kin T Murdoch T Richer B McGhee-Wilson D Ryan EA Shapiro JA Lakey JR The standardization of pancreatic donors for islet isolations Transplantation 2005 80 801 6 16210968 10.1097/01.tp.0000172216.47547.d5

[B4] Toyama H Takada M Suzuki Y Kuroda Y Activation of macrophage-associated molecules after brain death in islets Cell Transplant 2003 12 27 32 10.3727/000000003783985205 12693661

[B5] Contreras JL Eckstein C Smyth CA Sellers MT Vilatoba M Bilbao G Rahemtulla FG Young CJ Thompson JA Chaudry IH Eckhoff DE Brain death significantly reduces isolated pancreatic islet yields and functionality in vitro and in vivo after transplantation in rats Diabetes 2003 52 2935 42 10.2337/diabetes.52.12.2935 14633854

[B6] Ling Z Van de Casteele M Eizirik DL Pipeleers DG Interleukin-1beta-induced alteration in a beta-cell phenotype can reduce cellular sensitivity to conditions that cause necrosis but not to cytokine-induced apoptosis Diabetes 2000 49 340 5 10.2337/diabetes.49.3.340 10868954

[B7] Bertuzzi F Ricordi C Prediction of clinical outcome in islet allotransplantation Diabetes Care 2007 30 410 7 10.2337/dc06-1233 17259521

[B8] Sakuma Y Ricordi C Miki A Yamamoto T Pileggi A Khan A Alejandro R Inverardi L Ichii H Factors that affect human islet isolation Transplant Proc 2008 40 343 5 10.1016/j.transproceed.2007.12.019 18374062 PMC2614209

[B9] Melzi R Piemonti L Nano R Clissi B Calori G Antonioli B Marzorati S Perseghin G Di Carlo V Bertuzzi F Donor and isolation variables associated with human islet monocyte chemoattractant protein-1 release Transplantation 2004 78 1564 7 10.1097/01.TP.0000144184.20085.41 15599324

[B10] Uematsu S Akira S Toll-like receptors and innate immunity J Mol Med 2006 84 712 25 10.1007/s00109-006-0084-y 16924467

[B11] Iwasaki A Medzhitov R Toll-like receptor control of the adaptive immune responses Nat Immunol 2004 5 987 95 10.1038/ni1112 15454922

[B12] Jiang Z Georgel P Du X Shamel L Sovath S Mudd S Huber M Kalis C Keck S Galanos C Freudenberg M Beutler B CD14 is required for MyD88-independent LPS signaling Nat Immunol 2005 6 565 70 10.1038/ni1207 15895089

[B13] Hijiya N Miyake K Akashi S Matsuura K Higuchi Y Yamamoto S Possible involvement of toll-like receptor 4 in endothelial cell activation of larger vessels in response to lipopolysaccharide Pathobiology 2002 70 18 25 10.1159/000066000 12415188

[B14] Erridge C Burdess A Jackson AJ Murray C Riggio M Lappin D Milligan S Spickett CM Webb DJ Vascular cell responsiveness to Toll-like receptor ligands in carotid atheroma Eur J Clin Invest 2008 38 713 20 10.1111/j.1365-2362.2008.02010.x 18837796

[B15] Tsukumo DM Carvalho-Filho MA Carvalheira JB Prada PO Hirabara SM Schenka AA Araujo EP Vassallo J Curi R Velloso LA Saad MJ Loss-of-function mutation in Toll-like receptor 4 prevents diet-induced obesity and insulin resistance Diabetes 2007 56 1986 98 10.2337/db06-1595 17519423

[B16] Nicola JP Velez ML Lucero AM Fozzatti L Pellizas CG Masini-Repiso AM Functional Toll-Like Receptor 4 Conferring Lipopolysaccharide Responsiveness Is Expressed in Thyroid Cells Endocrinology 2008 18787027 10.1210/en.2008-0345

[B17] Hirata T Osuga Y Hirota Y Koga K Yoshino O Harada M Morimoto C Yano T Nishii O Tsutsumi O Taketani Y Evidence for the presence of toll-like receptor 4 system in the human endometrium J Clin Endocrinol Metab 2005 90 548 56 10.1210/jc.2004-0241 15509642

[B18] Wolf G Bohlender J Bondeva T Roger T Thaiss F Wenzel UO Angiotensin II upregulates toll-like receptor 4 on mesangial cells J Am Soc Nfephrol 2006 17 1585 93 10.1681/ASN.2005070699 16675600

[B19] Vitseva OI Tanriverdi K Tchkonia TT Kirkland JL McDonnell ME Apovian CM Freedman J Gokce N Inducible Toll-like receptor and NF-kappaB regulatory pathway expression in human adipose tissue Obesity (Silver Spring) 2008 16 932 7 10.1038/oby.2008.25 18292749 PMC3264059

[B20] Vives-Pi M Somoza N Fernandez-Alvarez J Vargas F Caro P Alba A Gomis R Labeta MO Pujol-Borrell R Evidence of expression of endotoxin receptors CD14, toll-like receptors TLR4 and TLR2 and associated molecule MD-2 and of sensitivity to endotoxin (LPS) in islet beta cells Clin Exp Immunol 2003 133 208 18 10.1046/j.1365-2249.2003.02211.x 12869026 PMC1808777

[B21] Parnaud G Bosco D Berney T Pattou F Kerr-Conte J Donath MY Bruun C Mandrup-Poulsen T Billestrup N Halban PA Proliferation of sorted human and rat beta cells Diabetologia 2008 51 91 100 10.1007/s00125-007-0855-1 17994216

[B22] Dasu MR Devaraj S Zhao L Hwang DH Jialal I High glucose induces toll-like receptor expression in human monocytes: mechanism of activation Diabetes 2008 57 3090 8 10.2337/db08-0564 18650365 PMC2570406

[B23] Goldberg A Parolini M Chin BY Czismadia E Otterbein LE Bach FH Wang H Toll-like receptor 4 suppression leads to islet allograft survival Faseb J 2007 21 2840 8 10.1096/fj.06-7910com 17475921

[B24] Kiely A Robinson A McClenaghan NH Flatt PR Newsholme P Toll-like receptor agonist induced changes in clonal rat BRIN-BD11 beta-cell insulin secretion and signal transduction J Endocrinol 2009 202 365 73 10.1677/JOE-09-0160 19553279

[B25] Ghanim H Mohanty P Deopurkar R Sia CL Korzeniewski K Abuaysheh S Chaudhuri A Dandona P Acute modulation of toll-like receptors by insulin Diabetes Care 2008 31 1827 31 10.2337/dc08-0561 18556339 PMC2518353

[B26] Tsan MF Gao B Cytokine function of heat shock proteins Am J Physiol Cell Physiol 2004 286 C739 44 10.1152/ajpcell.00364.2003 15001423

[B27] Ohashi K Burkart V Flohe S Kolb H Cutting edge: heat shock protein 60 is a putative endogenous ligand of the toll-like receptor-4 complex J Immunol 2000 164 558 61 10623794 10.4049/jimmunol.164.2.558

[B28] Warger T Hilf N Rechtsteiner G Haselmayer P Carrick DM Jonuleit H von Landenberg P Rammensee HG Nicchitta CV Radsak MP Schild H Interaction of TLR2 and TLR4 ligands with the N-terminal domain of Gp96 amplifies innate and adaptive immune responses J Biol Chem 2006 281 2254 53 10.1074/jbc.M502900200 16754684

[B29] Okamura Y Watari M Jerud ES Young DW Ishizaka ST Rose J Chow JC Strauss JF The extra domain A of fibronectin activates Toll-like receptor 4 J Biol Chem 2001 276 10229 33 10.1074/jbc.M100099200 11150311

[B30] Taylor KR Yamasaki K Radek KA Di Nardo A Goodarzi H Golenbock D Beutler B Gallo RL Recognition of hyaluronan released in sterile injury involves a unique receptor complex dependent on Toll-like receptor 4, CD44, and MD-2 J Biol Chem 2007 282 18265 75 10.1074/jbc.M606352200 17400552

[B31] Hodgkinson CP Laxton RC Patel K Ye S Advanced glycation end-product of low density lipoprotein activates the toll-like 4 receptor pathway implications for diabetic atherosclerosis Arterioscler Thromb Vasc Biol 2008 28 2275 81 10.1161/ATVBAHA.108.175992 18818414

[B32] Figueiredo RT Fernandez PL Mourao-Sa DS Porto BN Dutra FF Alves LS Oliveira MF Oliveira PL Graca-Souza AV Bozza MT Characterization of heme as activator of Toll-like receptor 4 J Biol Chem 2007 282 20221 9 10.1074/jbc.M610737200 17502383

[B33] Ricordi C Lacy PE Finke EH Olack BJ Scharp DW Automated method for isolation of human pancreatic islets Diabetes 1988 37 413 20 10.2337/diabetes.37.4.413 3288530

[B34] Ichii H Inverardi L Pileggi A Molano RD Cabrera O Caicedo A Messinger S Kuroda Y Berggren PO Ricordi C A novel method for the assessment of cellular composition and beta-cell viability in human islet preparations Am J Transplant 2005 5 1635 45 10.1111/j.1600-6143.2005.00913.x 15943621

[B35] Pfaffl MW A new mathematical model for relative quantification in real-time RT-PCR Nucleic Acids Res 2001 29 e45 10.1093/nar/29.9.e45 11328886 PMC55695

